# P-714. Symptoms Associated with High Viral Loads of Common Cold Coronaviruses in Hematopoietic Cell Transplantation Recipients

**DOI:** 10.1093/ofid/ofae631.910

**Published:** 2025-01-29

**Authors:** Chikara Ogimi, Faduma A Abukar, Alpana waghmare, Angela P Campbell, Keith R Jerome, Filippo Milano, Guang-Shing Cheng, Janet A Englund, Michael J Boeckh

**Affiliations:** National Center for Child Health and Development, Setagaya-ku, Tokyo, Japan; Fred Hutch Cancer Center, TUKWILA, Washington; Fred Hutchinson Cancer Center; Seattle Children's Hospital, Seattle, Washington; Centers for Disease Control and Prevention, Atlanta, GA; Fred Hutchinson Cancer Center, Seattle, Washington; Fred Hutchinson Cancer Center, Seattle, Washington; University of Washington; Fred Hutchinson Cancer Center, Seattle, Washington; Seattle Children’s Hospital, Seattle, Washington; Fred Hutchinson Cancer Center, Seattle, Washington

## Abstract

**Background:**

Limited data exist on the association between specific symptoms and viral loads of common cold coronavirus (ccCOV). We investigated this potential association in allogeneic hematopoietic cell transplant (HCT) recipients.

**Figure 1.**
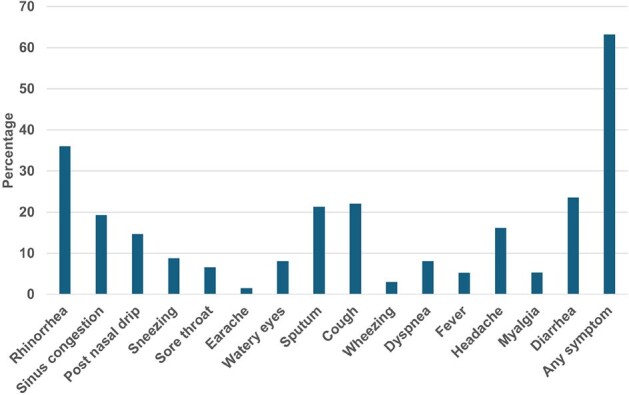
Proportion of sampling time points with each symptom

**Methods:**

Four hundred seventy-one children and adults were prospectively followed with weekly symptom questionnaires through one year after allogeneic HCT at the Fred Hutchinson Cancer Center (12/2005-2/2010). Multiplex PCR for 11 respiratory viruses was performed on combined nasal wash and throat swab samples collected weekly through day 100 post-HCT, and then every three months or if symptoms were present through one-year post-HCT. Positive samples were evaluated for viral loads with quantitative real-time PCR (copies/ml). We included time points with the detection of ccCOV only and corresponding 15-point symptom survey data available post-HCT. To assess the association between specific symptoms and viral loads, the Mann-Whitney or Jonckheere-Terpstra tests were used to compare 2 or >2 groups for continuous variables, respectively.

**Figure 2.**
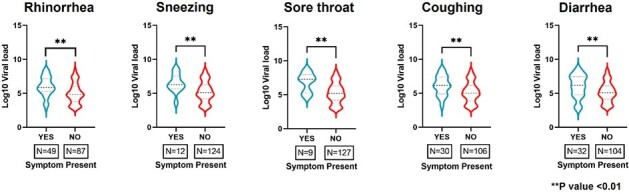
Symptoms associated with higher viral loads

**Results:**

Among 6,276 samples tested, 251 samples (79 patients) were positive for ccCoV. One hundred thirty-six sampling time points (52 patients) met the inclusion criteria. The proportion of sample time points with each symptom present is shown in **Figure 1**. When we compared viral loads with the presence of each of the 15 symptoms, higher viral loads were associated with the presence of four respiratory tract symptoms (rhinorrhea, sneezing, sore throat, and coughing) and one systemic symptom (diarrhea) (p< 0.01 for each) (**Figure 2**). Viral loads appeared to increase with increasing number of respiratory tract symptoms (p< 0.001) (**Figure 3**).

**Figure 3.**
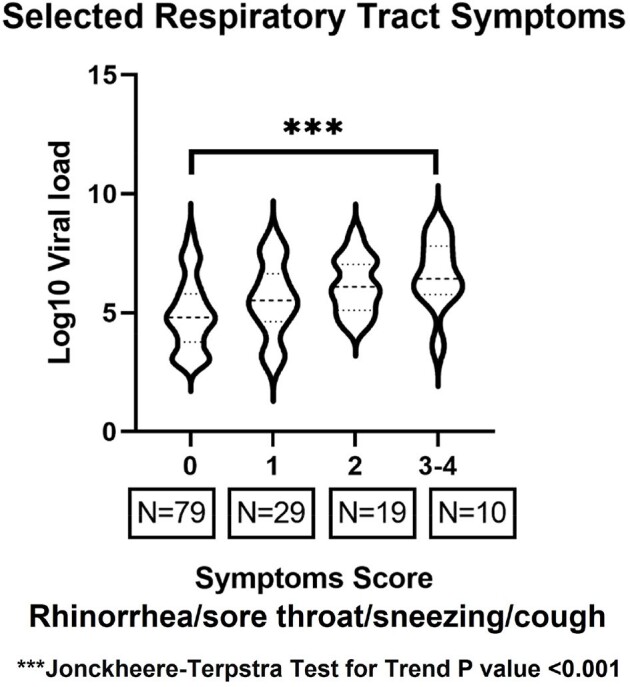
Viral loads compared with the number of respiratory tract symptoms

**Conclusion:**

The presence of specific respiratory tract symptoms is associated with higher viral loads of ccCOV in the upper respiratory tract in allogeneic HCT recipients. The association with diarrhea suggests the possibility of fecal shedding, but this was not evaluated in our study. Patients with specific symptoms may be more likely to transmit virus given their higher viral loads. Studies of these associations for other respiratory viruses are warranted.

**Disclosures:**

**Chikara Ogimi, MD, PhD**, AstraZeneca: Honoraria|bioMerieux Japan Ltd.: Honoraria|ELSEVIER: Honoraria|KYORIN: Honoraria|Miyarisan: Honoraria|MSD: Honoraria|NOVARTIS: Honoraria|Pfizer: Honoraria **Alpana waghmare, MD**, Allovir: Grant/Research Support|Ansun Biopharma: Grant/Research Support|GlaxoKlineSmith: Advisor/Consultant|GlaxoKlineSmith: Grant/Research Support|Pfizer: Grant/Research Support|Vir: Advisor/Consultant **Janet A. Englund, MD**, Abbvie: Advisor/Consultant|AstraZeneca: Advisor/Consultant|AstraZeneca: Grant/Research Support|GlaxoSmithKline: Advisor/Consultant|GlaxoSmithKline: Grant/Research Support|Meissa Vaccines: Advisor/Consultant|Merck: Advisor/Consultant|Pfizer: Board Member|Pfizer: Grant/Research Support|Pfizer: Speaker at meeting|SanofiPasteur: Advisor/Consultant|Shinogi: Advisor/Consultant **Michael J. Boeckh, MD PhD**, Allovir: Advisor/Consultant|Allovir: Grant/Research Support|AstraZeneca: Advisor/Consultant|AstraZeneca: Grant/Research Support|Merck: Advisor/Consultant|Merck: Grant/Research Support|Moderna: Advisor/Consultant|Moderna: Grant/Research Support|Symbio: Advisor/Consultant

